# Supplementary material for: Comprehensive Annotation and Functional Exploration of MicroRNAs in Lettuce
Source: Front Plant Sci. 2021 Dec 24;12:781836. doi: 10.3389/fpls.2021.781836 (PMC8739914; doi:10.3389/fpls.2021.781836)
Supplement: Supplementary file 3 [file Data_Sheet_9.ZIP › GSM2230752.plot/Lsa-miR1446_Lsat_1_v5_gn_8_90101.1_650_TPlot.pdf]

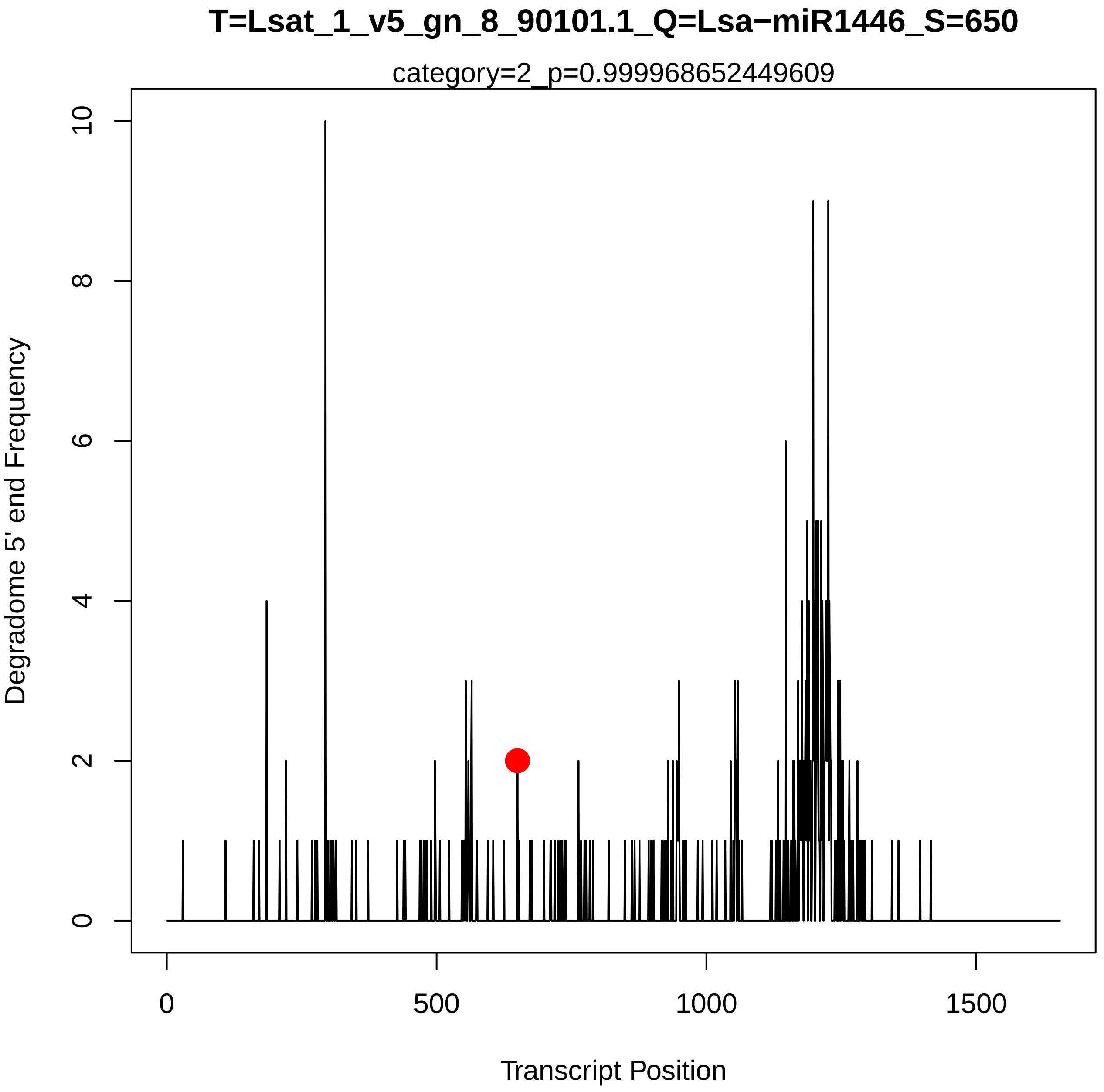

T=Lsat_1_v5_gn_8_90101.1_Q=Lsa−miR1446_S=650
category=2_p=0.999968652449609
Degradome 5' end Frequency
0
2
4
6
8
10
0
500
1000
1500
Transcript Position
